# The Risk of SARS-CoV-2 Transmission in Community Indoor Settings: A Systematic Review and Meta-analysis

**DOI:** 10.1093/infdis/jiae261

**Published:** 2024-05-16

**Authors:** Mark Rohit Francis, Saheed Gidado, J Pekka Nuorti

**Affiliations:** Health Sciences Unit, Faculty of Social Sciences, Tampere University, Tampere, Finland; Health Sciences Unit, Faculty of Social Sciences, Tampere University, Tampere, Finland; Health Sciences Unit, Faculty of Social Sciences, Tampere University, Tampere, Finland; Infectious Diseases and Vaccinations Unit, Department of Health Security, Finnish Institute for Health and Welfare, Helsinki, Finland

**Keywords:** SARS-CoV-2, COVID-19, transmission, indoors, community settings, secondary attack rate

## Abstract

**Background:**

Quantifying the risk of severe acute respiratory syndrome coronavirus 2 (SARS-CoV-2) transmission in indoor settings is crucial for developing effective non-vaccine prevention strategies and policies. However, summary evidence on the transmission risks in settings other than households, schools, elderly care, and health care facilities is limited. We conducted a systematic review to estimate the secondary attack rates (SARs) of SARS-CoV-2 and the factors modifying transmission risk in community indoor settings.

**Methods:**

We searched Medline, Scopus, Web of Science, WHO COVID-19 Research Database, MedrXiv, and BiorXiv from 1 January 2020 to 20 February 2023. We included articles with original data for estimating SARS-CoV-2 SARs. We estimated the overall and setting-specific SARs using the inverse variance method for random-effects meta-analyses.

**Results:**

We included 34 studies with data on 577 index cases, 898 secondary cases, and 9173 contacts. The pooled SAR for community indoor settings was 20.4% (95% confidence interval [CI], 12.0%–32.5%). The setting-specific SARs were highest for singing events (SAR, 44.9%; 95% CI, 14.5%–79.7%), indoor meetings and entertainment venues (SAR, 31.9%; 95% CI, 10.4%–65.3%), and fitness centers (SAR, 28.9%; 95% CI, 9.9%–60.1%). We found no difference in SARs by index case, viral, and setting-specific characteristics.

**Conclusions:**

The risk of SARS-CoV-2 transmission was highest in indoor settings where singing and exercising occurred. Effective mitigation measures such as assessing and improving ventilation should be considered to reduce the risk of transmission in high-risk settings. Future studies should systematically assess and report the host, viral, and setting-specific characteristics that may modify the transmission risks of SARS-CoV-2 and other respiratory viruses in indoor environments.

## Teaser

We conducted a systematic review and meta-analysis to estimate the risk of SARS-CoV-2 transmission in community indoor settings. We found that risk was greatest in settings where singing and exercising occurred and recommend implementing effective mitigation measures in these settings.

The coronavirus disease 2019 (COVID-19) pandemic has resulted in over 770 million confirmed cases and nearly 7 million deaths by December 2023 [1]. In the early stages of the pandemic, households were identified as the major setting for severe acute respiratory syndrome coronavirus 2 (SARS-CoV-2) transmission [2, 3]. However, large infection clusters were also reported in other settings, such as schools, hospitals, elderly care facilities, religious gatherings, food processing plants, and shopping centers [4]. While the risk of SARS-CoV-2 transmission is higher in households than in workplaces and social settings (religious, shopping, and entertainment venues), it is vital to understand which settings and behaviors are associated with increased risk of infection clusters [4, 5]. Updated evidence on the risk of transmission in community settings can enable more effective targeting of public health interventions to minimize the impact of future outbreaks caused by current and emerging SARS-CoV-2 variants.

There is considerable evidence in indoor settings that SARS-CoV-2 spreads primarily by short- and long-range airborne transmission through respiratory aerosols [6–8]. A recent systematic review reported the potential for long-distance airborne transmission in restaurants, workplaces, and choir venues, pointing to insufficient air replacement as a primary driver of transmission [8]. In addition to host factors such as age, infectiousness, and severity of illness and viral factors, environmental factors like ventilation, preventive measures (including mask-wearing, hand hygiene, and social distancing), and contact patterns (the proximity of occupants, exposure duration, contact frequency, and type of activity) can play an important role in SARS-CoV-2 transmission in indoor settings [6, 9]. Understanding the key drivers of transmission in community indoor settings can help develop better prevention and control measures.

Although vaccines are the most effective prevention measures for severe illness, they do not substantially reduce SARS-CoV-2 transmission in crowded settings and against newer variants [10–13]. Our study, therefore, focuses on the added benefit of accounting for different setting types and characteristics to improve the interpandemic prevention of SARS-CoV-2 and other respiratory pathogens. We conducted a systematic review and meta-analysis to estimate the secondary attack rate (SAR) of SARS-CoV-2, including the factors modifying the risk of transmission in community indoor settings. These settings broadly included workplaces, restaurants, bars, nightclubs, shopping and fitness centers, religious gatherings, and sporting and singing events.

## METHODS

### Data Sources and Search Strategy

We searched the following electronic databases: Medline (via Ovid), Scopus, Web of Science, World Health Organization COVID-19 Research Database, and preprint servers MedrXiv and BiorXiv (via iSearch COVID-19 portfolio). Our search strategy included 3 concepts: “SARS-CoV-2/COVID-19” (15 search terms), “transmission” (13 terms), and “setting” (94 terms) (Supplementary Material 1). Boolean operators “OR” was used between terms and “AND” between the concepts. Medical subject headings (MeSH terms) were used to maximize the number of studies retrieved when available. The search duration was from 1 January 2020 to 20 February 2023.

In addition, we hand-searched the reference lists of the included articles and existing systematic reviews on the topic for more eligible studies. The review protocol was registered at PROSPERO before the database searches were initiated (CRD42022362920). Our systematic review follows the Preferred Reporting Items for Systematic Reviews and Meta-Analyses (PRISMA) 2020 guidelines (Supplementary Material 2).

### Eligibility Criteria

We included articles (published articles, accepted manuscripts, and preprints) with original data for estimating the SAR of SARS-CoV-2. The articles should have reported either the SAR in specific indoor settings or the data required to calculate the SARs among close contacts (ie, the number of infected contacts and the total number of contacts). All observational studies, such as outbreak investigations, contact-tracing studies, and other epidemiological investigations (cohort, case-control, and cross-sectional studies) were included.

We excluded descriptive case reports, seroprevalence studies not describing SARS-CoV-2 transmission risks, studies on transmission in households, schools and daycares, health care, and long-term care and nursing facilities, modeling studies, ecological studies, biological studies, environmental sampling studies, intervention studies, studies on vertical transmission, commentaries or opinion pieces, conference abstracts, and studies in languages other than English.

### Study Selection and Data Extraction

We imported the records retrieved from each database to Zotero for deduplication, after which 2 investigators (M. R. F. and S. G.) conducted study screening and selection using Rayyan, an online screening tool [14]. Records were screened by titles and abstracts, followed by the full texts according to the eligibility criteria. Any disagreements were resolved through consensus.

Two investigators (M. R. F. and S. G.) extracted data from the included studies using prepiloted Google Forms questionnaires. We collected data about the first author, study location and design, investigation dates, setting type and characteristics (dimensions, ventilation, activity type, duration of activity, and mitigation measures), number of index cases, contacts, and secondary cases, testing strategy (universal or symptomatic) and method (reverse transcription polymerase chain reaction [RT-PCR] or antigen tests), follow-up duration, and number of tests per contact. We also extracted the age, sex, and vaccination status of index cases and contacts, predominant SARS-CoV-2 variant, and community COVID-19 incidence, when available.

### Study Definitions

The index case definitions came from the studies themselves and were either the first case to be confirmed or the confirmed case with the earliest date of symptom onset in a given setting [2]. The definitions for contacts were also from the studies; contacts were broadly defined as people who shared an indoor space with an index case for any length of time and were followed up and tested either by RT-PCR or antigen tests (Supplementary Table 1). The SAR was defined as the percentage of contacts of an index case that tested positive for SARS-CoV-2 by RT-PCR or antigen tests in specific community indoor settings.

### Quality Assessment

Two investigators (M. R. F. and S. G.) independently assessed the methodological quality of all included studies using a modified version of the Newcastle-Ottawa Quality Assessment Scale for Observational Studies [2, 15]. The modified tool contained 7 questions, focusing on the representativeness and definition of index cases, the total number of contacts, and the testing strategy and duration of follow-up of contacts. We did not assess the representativeness of the index cases as our review focused on studies reporting secondary transmission within specific community indoor settings. Based on the 6 remaining questions, studies could get a maximum score of 7 points and were classified as high quality (5–7 points), moderate quality (3–4 points), or low quality (1–2 points) using previously reported cutoffs [2] (Supplementary Table 2).

### Statistical Analysis

All included studies were eligible for meta-analysis. We estimated the pooled SARs and 95% confidence intervals (CIs) with random effects meta-analyses using the inverse variance method and restricted maximum likelihood estimator to account for the heterogeneity between studies [16, 17]. The SARs were transformed with the logit function before pooling the study-level estimates, and exact binomial 95% CIs were calculated using the Clopper-Pearson method [18]. We recalculated the study-level SARs, their associated standard errors, and 95% CIs and presented back-transformed inverse logit values. We accounted for clustering within the individual studies (as some studies reported transmission in more than 1 setting) by adding an additional level for the setting to the random effects models.

We stratified the SARs into the following setting categories: workplaces, dining settings, fitness centers, religious settings, singing events, and other community settings (including indoor meetings, a chalet, a bathing pool, and nightclubs). Prespecified (index case symptom status and age, contact testing strategy, setting dimensions, duration of exposure, ventilation type, masking, and risk of bias) and post hoc (study design, geographic location, and duration of follow-up of contacts) subgroup analyses were done using mixed-effects models. These models utilized random effects to model the variability in the study estimates within each subgroup and fixed effects to model the differences in effects across subgroups [19]. We omitted studies with missing information from the subgroup analyses and applied the Hartung-Knapp adjustment for small study sizes to all the test statistics and 95% CIs [20].

As sensitivity analyses, we excluded contacts that were identified but not tested, non–laboratory-confirmed secondary cases, studies with a single index case (to minimize possible biases from single-case studies) [5], low- and moderate-quality studies, and preprints to assess whether their removal impacted the summary estimates. We assessed small-study effects by plotting sample size (the total number of contacts) against logit-transformed SARs, as conventional funnel plots and publication bias tests are known to be erroneous when pooling proportions [21]. Heterogeneity between studies was evaluated using the *I^2^* test statistic. We cleaned the extracted data using Stata (version 18) and utilized the *metafor* package in R (version 4.3.0) for the meta-analyses and forest plots.

## RESULTS

### Search Results

**Figure 1.**
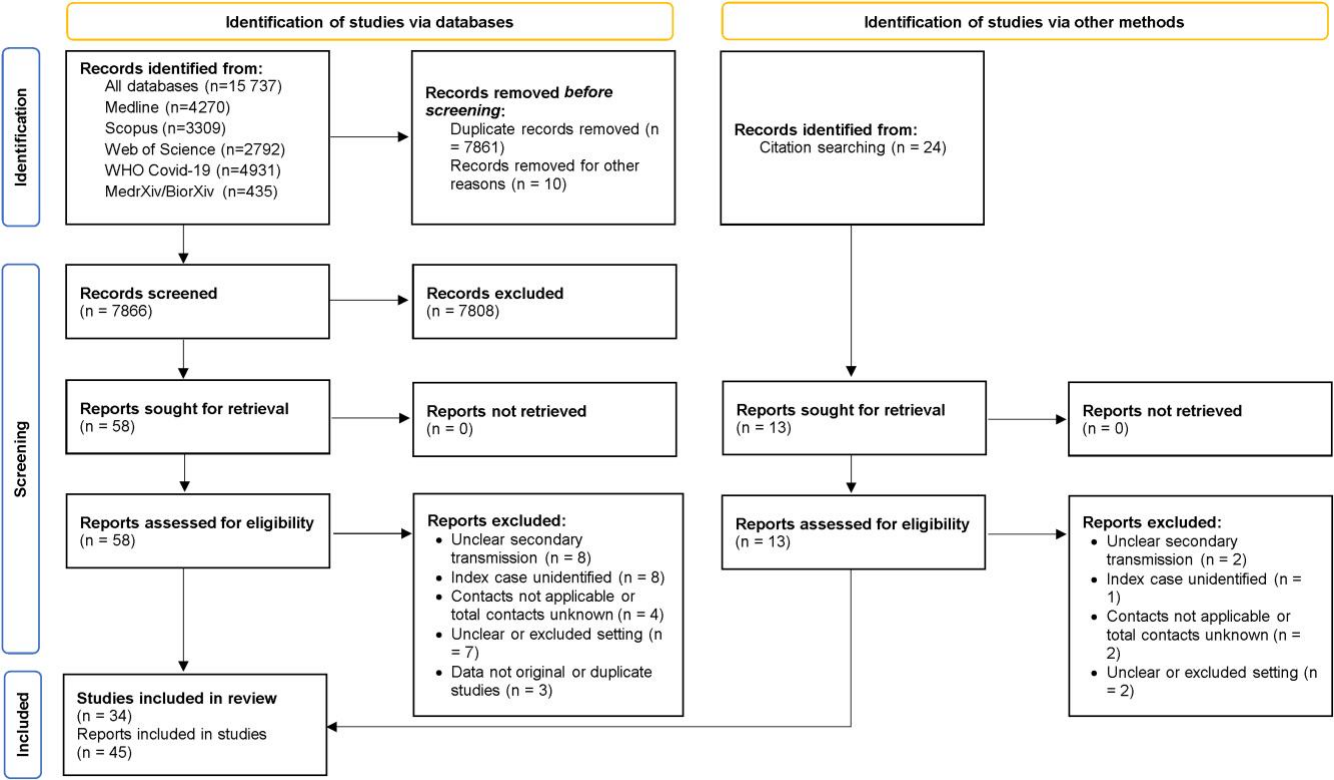
Preferred Reporting Items for Systematic reviews and Meta-Analyses (PRISMA) flow diagram for study selection.

Systematic search of the electronic databases and preprint servers identified 15 737 studies (Figure 1). Of these, 7861 were duplicates, resulting in 7866 studies eligible for screening. Following the title and abstract screening, we evaluated 58 full texts; 28 studies met our inclusion criteria. We identified 24 studies by citation searching and evaluated 13 full texts, adding 6 more studies to the review (Figure 1 and Supplementary Table 3). Thirty-four studies containing data on 45 transmission events were included in our review.

### Study Characteristics

Of the included studies, 32 were published and 2 were preprints. The studies were primarily outbreak investigations (n = 22), and the rest were contact-tracing (n = 7), cross-sectional (n = 2), and cohort (n = 3) studies (Table 1). All the studies were conducted between January 2020 and December 2021, when the dominant SARS-COV-2 variants were Wuhan wild type (n = 30), Delta (n = 2), and Omicron (n = 2).

**Table 1.** Characteristics of the Included Studies Reporting on SARS-CoV-2 Transmission in Community Indoor Settings, 1 January 2020 to 20 February 2023, 34 Studies, 45 Transmission Events

Author (Year Published	Location	Study Design (Time Period)	Study Setting (Specification)	Contact Testing Strategy	Contact Testing Method	Index Cases	Contacts Traced/Tested	Secondary Cases
Bae et al (2020) [22]	Cheonan, Korea	Outbreak investigation (24 February–13 March 2020)	Fitness centers	Symptomatic testing	RT-PCR	8	233/Not specified	57
Bao et al (2021) [23]	China	Outbreak investigation (18 January–6 February 2020)	Other setting (entertainment venue)	Unclear	RT-PCR	1	56/56	15
Brandal et al (2021) [24]	Oslo, Norway	Cohort study (26 November–4 December 2021)	Dining setting (party in a restaurant)	Universal testing	RT-PCR, WGS	1	116/110	80
Charlotte et al (2023) [25]	France	Outbreak investigation (9 May–20 June 2020)	Singing event (choir rehearsal)	Unclear	Unclear	3	27/Not specified	19
Chaw et al (2020) [26]	Brunei	Contact tracing study (March 2020)	Religious setting (not specified)	Universal testing	RT-PCR	19	54/54	8
			Workplaces (not specified)	Universal testing	RT-PCR	19	848/848	6
Cheng et al (2022) [27]	Hong Kong, China	Outbreak investigation (December 2021)	Workplace (restaurant staff)	Universal testing	RT-PCR	1	22/22	0
			Dining setting (restaurant customers)	Universal testing	RT-PCR	1	207/207	6
Danis et al (2020) [28]	Contamines-Montjoie, France	Contact tracing study (February 2020)	Other setting (chalet)	Universal testing	RT-PCR	1	15/15	11
Dougherty et al (2021) [29]	Oklahoma, USA	Outbreak investigation (15 April–3 May 2021)	Fitness center (gym)	Unclear	Unclear	1	20/20	2
Groves et al (2021) [30]	Hawaii, USA	Outbreak investigation (29 June–11 July 2020)	Fitness center	Symptomatic testing	RT-PCR	1	14/14	10
Hamner et al (2020) [31]	Washington, USA	Outbreak investigation (March 2020)	Singing event (choir rehearsal)	Universal testing	RT-PCR	1	60/60	52
Hijnen et al (2020) [32]	Munich, Germany	Outbreak investigation (February 2020)	Other setting (scientific meeting)	Universal testing	RT-PCR	1	13/11	11
James et al (2020) [33]	Arkansas, USA	Outbreak investigation (6 March–23 March 2020)	Religious setting (church services)	Symptomatic testing	Unclear	2	90/45	33
Jang et al (2020) [34]	Cheonan, South Korea	Contact tracing study (15 February–9 March 2020)	Fitness centers	Universal testing	RT-PCR	6	217/217	57
Katelaris et al (2021) [35]	Sydney, Australia	Outbreak investigation (18 June–27 June 2020)	Singing event (choir singing)	Universal testing	RT-PCR	1	508/434	12
Lam et al (2021) [36]	Hong Kong, China	Outbreak investigation (10 February–22 February 2020)	Dining setting (restaurant)	Symptomatic testing	RT-PCR	1	28/5	5
Montecucco et al (2021) [37]	Genoa, Italy	Cross-sectional study (October 2020–March 2021)	Workplace (offices)	Unclear	RT-PCR	13	53/53	1
			Dining setting (shared eating areas)	Unclear	RT-PCR	6	47/47	9
			Workplace (classrooms)	Unclear	RT-PCR	5	91/91	2
Moreno et al (2021) [38]	United States	Outbreak investigation (September 2020)	Other setting (indoor meeting)	Universal testing	RT-PCR, Antigen tests	1	10/10	4
Muller et al (2021) [39]	Berlin, Germany	Contact tracing study (March 2020)	Other setting (nightclub)	Symptomatic testing	RT-PCR, Antigen tests	1	311/Not specified	39
Ng et al (2020) [40]	Singapore	Cohort study (23 January–3 April 2020)	Workplaces (not specified)	Symptomatic testing	RT-PCR	225	2231/Not specified	30
Noman et al (Unpublished)	Chattogram, Bangladesh	Contact tracing study (July–September 2020)	Religious setting (funeral)	Universal testing	RT-PCR	22	79/79	2
			Workplaces (not specified)	Universal testing	RT-PCR	77	177/177	7
Nsekuye et al (2021) [41]	Rwanda	Outbreak investigation (14 March–22 April 2020)	Other setting (nightclub)	Universal testing	RT-PCR	1	264/173	9
Park et al (2020) [42]	Seoul, South Korea	Outbreak investigation (9 March–21 March 2020)	Workplace (office)	Universal testing	RT-PCR	1	27/27	2
			Workplace (call center)	Universal testing	RT-PCR	1	216/216	94
Pauser et al (2021) [43]	Germany	Cross-sectional study (November 2020)	Fitness centers (sporting facility)	Universal testing	RT-PCR	1	68/61	35
Sarti et al (2021) [44]	Italy	Outbreak investigation (20 November–7 December 2020)	Workplace (office)	Universal testing	RT-PCR	1	4/4	4
Seok et al (2022) [45]	Gyeonggi-do, Korea	Cohort study (1 January–30 April 2020)	Workplaces (not specified)	Universal testing	RT-PCR	28	377/377	32
			Religious settings (not specified)	Universal testing	RT-PCR	53	483/483	65
			Fitness centers (not specified)	Universal testing	RT-PCR	3	45/45	0
Shah et al (Preprint) [46]	Netherlands	Outbreak investigation (September–October 2020)	Singing event (choir rehearsal)	Symptomatic testing	RT-PCR	1	13/Not specified	6
			Singing event (choir rehearsal)	Symptomatic testing	RT-PCR	1	8/Not specified	5
			Singing event (choir singing)	Symptomatic testing	RT-PCR	1	14/Not specified	7
Shen et al (2020) [47]	Jiaxing city, China	Outbreak investigation (January 2020)	Dining setting (restaurant)	Symptomatic testing	RT-PCR	1	7/Not specified	2
Shin et al (2022) [48]	North Gyeongsang, Korea	Outbreak investigation (January 2021)	Fitness center (gym)	Universal testing	RT-PCR	1	108/108	6
Sundar et al (2021) [49]	Chennai, India	Contact tracing study (1 August–20 August 2020)	Workplace (indoor spaces)	Universal testing	RT-PCR	18	169/Not specified	44
Tian et al (2021) [50]	Liaocheng city, China	Outbreak investigation (15 January–22 January 2020)	Workplace (supermarket)	Universal testing	RT-PCR	1	120/120	11
Yusef et al (2020) [51]	Jordan	Outbreak investigation (March 2020)	Religious setting (wedding)	Universal testing	RT-PCR	1	350/350	76
Zhang et al (2020) [52]	Guangzhou, China	Contact tracing study (28 January–15 March 2020)	Workplaces (not specified)	Universal testing	Antigen tests	38	119/119	0
Zhang et al (2022) [53]	Guangzhou, China	Outbreak investigation (May 2021)	Dining setting (restaurant)	Universal testing	RT-PCR	1	26/Not specified	9
Zhou et al (2022) [54]	Wenzhou, China	Outbreak investigation (January 2020)	Workplace (store salespersons)	Symptomatic testing	RT-PCR	3	1254/Not specified	9
			Workplace (store administrators)	Symptomatic testing	RT-PCR	3	73/Not specified	4

Abbreviations: RT-PCR, reverse transcription polymerase chain reaction; WGS, whole genome sequencing.

Eight studies were conducted in China, 5 in the United States and South Korea, 3 in Germany, 2 in Italy and France, and 1 in Norway, the Netherlands, Jordan, Singapore, Brunei, Australia, Rwanda, India, and Bangladesh (Table 1). The included studies contained information on 577 index cases, 898 secondary cases, and 9173 contacts. The index cases (n = 30 studies) and secondary cases (n = 29) were predominantly ascertained by RT-PCR testing. Contact testing was generally conducted irrespective of symptoms (n = 21 studies) or through symptomatic testing (n = 9). Three studies [24, 43, 46] reported the immune status of index cases/contacts at baseline. The individuals in these studies were predominantly SARS-CoV-2 infection naive. The setting-specific characteristics potentially associated with the risk of SARS-CoV-2 transmission are listed in Supplementary Table 4 and described in Supplementary Material 3.

### Overall and Setting-Specific Secondary Attack Rates

The overall SAR across community indoor settings was 20.4% (95% CI, 12.0%–32.5%), with significant heterogeneity (*I*^2^ = 96.1%, *P* < .0001). The SAR, including only PCR-confirmed secondary cases, was 18.3% (95% CI, 11.1%–28.7%). Furthermore, the SAR restricting the total contacts to those tested was 22.5% (95% CI, 11.1%–40.2%).

**Figure 2.**
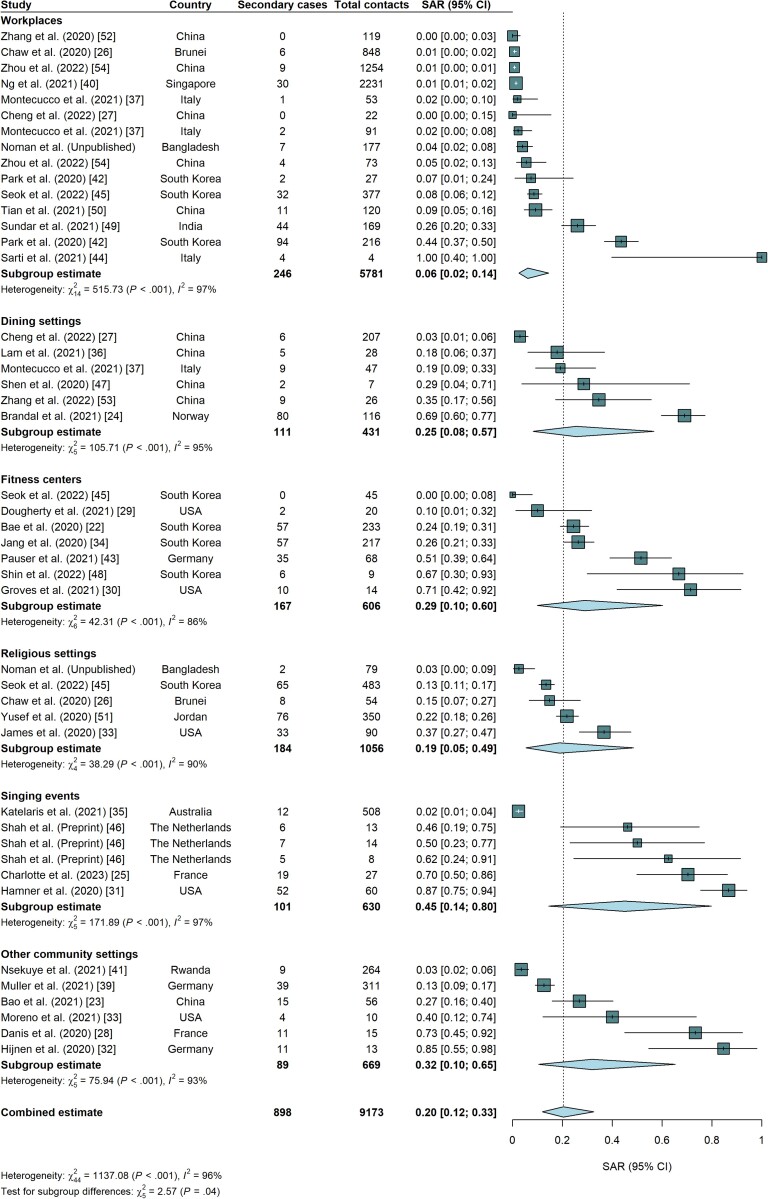
Forest plot for the overall and setting-specific secondary attack rates. Study-level estimates and exact binomial 95% CIs are shown along with the pooled summary SARs by setting category and across studies. The studies are arranged in ascending order of the magnitude of the study-level estimates within each setting category. Abbreviations: CI, confidence interval; SAR, secondary attack rate.

The SARs for workplaces (n = 15 transmission events) ranged from 0.0% (for workplaces in Guangzhou, China) [52] to 100.0% (in an office in Italy) [44], with a pooled estimate of 6.2% (95% CI, 2.5%–14.4%) (Figure 2). Upon stratifying the workplaces further, we estimated that the SAR for offices [37, 42, 44] was higher than the SAR for retail and unspecified workplaces (21.2% vs 3.1%, *P* = .046) [26, 27, 37, 40, 45, 49, 50, 52, 54].

The SAR for dining settings (n = 6) ranged from 2.9% (in a restaurant in Hong Kong) [27] to 69.0% (for a Christmas party in a restaurant in Oslo, Norway) [24], with a pooled estimate of 25.5% (95% CI, 8.2%–56.7%). The religious settings (n = 5) included church services [33], a wedding [51], a funeral [Noman et al unpublished], and 2 unspecified religious gatherings [26, 45], and had a pooled SAR of 18.8% (95% CI, 5.3%–48.6%). The SARs for singing events (n = 6) ranged from 2.4% (for singing events at a church in Sydney, Australia) [35] to 86.7% (for a choir rehearsal in Washington, United States) [31], with a pooled SAR of 44.9% (95% CI, 14.5%–79.7%). Three were outbreaks at singing events in the Netherlands with high SARs (46.2%–62.5%), as reported in a preprint [46].

The pooled SAR for fitness centers (n = 7) was 28.9% (95% CI, 9.9%–60.1%). The SARs for the other community indoor settings ranged from 3.4% (in a nightclub in Kigali, Rwanda) [41] to 84.6% (for a scientific meeting in a hotel in Munich, Germany) [32]. The pooled SAR for other community settings was 31.9% (95% CI, 10.4%–65.3%). The heterogeneity (*I*^2^) of the study-level SARs for the settings ranged from 85.8% (for fitness centers) to 97.3% (for workplaces). Nevertheless, there was a statistically significant difference between the setting-specific SARs (*P* = .042).

### Secondary Attack Rates Stratified by Study, Index Case, and Setting Characteristics

We did not find statistically significant differences in the SARs by study design, geographic location, circulating strain, testing strategy, the duration of follow-up of contacts, or the age and symptom status of index cases (Table 2). We also found no differences in the SARs by setting characteristics such as dimensions, ventilation, mask use, physical distancing, or the duration of exposure (Table 2).

**Table 2.** Subgroup Analysis of the Secondary Attack Rates by Study, Index Case, and Setting-Specific Characteristics

Characteristic	Number of Studies	Number of Events	Secondary Cases	Total Contacts	SAR, % (95% CI)^a^	*P* Value^c^
Study design						
Outbreak investigation	22	27	470	3673	26.9 (14.2–45.0)	.309
Contact tracing	7	9	174	1989	10.9 (2.9–33.0)	
Others	5	9	254	3511	13.4 (3.2–42.5)	
Geographic location						
China	8	10	61	1912	8.4 (2.5–25.2)	.175
South Korea	5	8	313	1607	23.7 (6.2–59.2)	
United States	5	5	101	194	49.2 (15.5–83.7)	
Others	16	22	423	5460	20.7 (9.7–38.9)	
Predominant circulating strain						
Wild type	30	40	801	8782	20.6 (11.6–34.0)	.927
Variant of concern (Delta/Omicron)	4	5	97	391	19.1 (3.4–61.1)	
Index case symptom status						
Asymptomatic	4	4	191	428	54.1 (16.4–87.6)	.162
Presymptomatic	6	6	33	200	34.9 (9.6–73.1)	
Symptomatic	20	29	573	5870	17.2 (8.7–31.2)	
Age of index case^b^						
< 40 y	8	11	191	5655	7.8 (2.5–21.6)	.324
40 y and above	6	9	231	1634	17.2 (4.7–46.9)	
Contact testing strategy						
Universal testing	20	27	643	4603	21.8 (10.7–39.6)	.757
Symptomatic testing	9	12	207	4276	18.1 (5.8–44.2)	
Duration of follow-up of contacts						
>14 d	4	4	149	1085	16.4 (2.5–60.2)	.311
14 d	16	23	403	6120	19.3 (8.3–39.0)	
<14 d	4	4	131	225	55.9 (13.8–90.9)	
Setting dimensions						
<100 m^2^	4	4	96	283	54.2 (12.9–90.5)	.702
100 m^2^ and above	4	7	108	384	41.2 (7.6–85.8)	
Duration of exposure						
≤ 2 h	4	6	112	627	39.7 (13.1–74.3)	.575
> 2 h	5	6	164	257	48.3 (19.3–78.5)	
Room ventilation^d^						
None/poor rating	6	7	60	1900	23.0 (3.7–69.9)	.937
Natural	1	2	11	21	28.2 (1.5–91.2)	
Mechanical	3	5	79	502	20.2 (2.2–74.4)	
Reported mask-wearing^e^						
Not required/low adherence	11	14	257	3412	32.5 (12.1–62.7)	.731
Required/high adherence	4	6	27	232	24.6 (3.7–73.6)	
Physical distancing^e^						
Not required/low adherence	4	4	192	773	35.9 (6.0–83.2)	.964
Required/high adherence	8	10	102	785	34.7 (9.4–73.0)	

Abbreviations: CI, confidence interval; SAR, secondary attack rate.

^a^The Hartung-Knapp adjustment for small study sizes was applied to all test statistics and 95% CIs.

^b^The mean age was used if there was >1 index case per setting.

^c^The *P* values are from the test for subgroup differences in the SARs.

^d^Natural ventilation included open doors and/or windows and mechanical ventilation included ventilation systems, air purifiers and filters. The rating of room ventilation was used if reported by the study investigators.

^e^Mask-wearing and physical distancing were categorized based on an existing recommendation and/or reported adherence in the settings of interest; Adherence was considered high if ≥ 65% (the median value) of individuals were reported to have worn masks or maintained distancing recommendations in each setting and low if not.

### Sensitivity Analyses and Publication Bias Assessment

**Figure 3.**
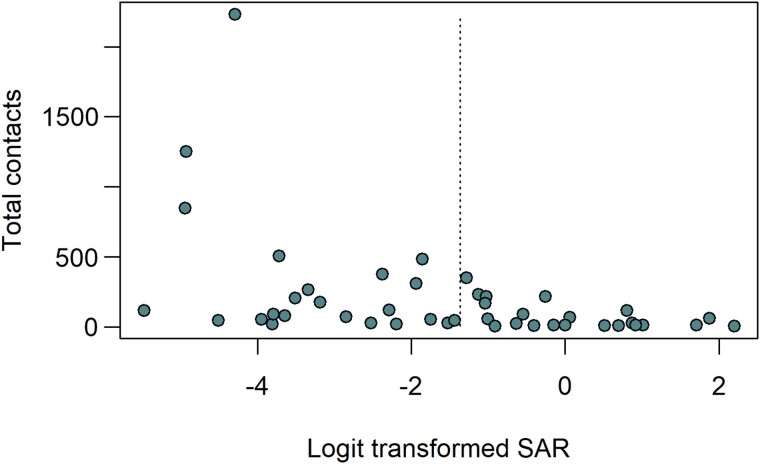
Funnel plot of study size (total contacts) versus logit transformed SARs to assess publication bias in the included studies, as recommended by Hunter et al (2014) [21]. Abbreviation: SAR, secondary attack rate.

In sensitivity analyses, we estimated that the pooled SAR for studies with >1 index case was 7.0% (95% CI, 3.2%–14.8%), and the pooled SAR excluding preprints [Noman et al unpublished, 46] was 20.0% (95% CI, 11.5%–32.6%). While exploring the potential for publication bias, we observed some asymmetry in the funnel plot for all included studies due to 4 outliers because of their sample size (Figure 3). These studies reported secondary transmission in workplaces and had low SARs (<1.5%) [26, 40, 52, 54]. Removing these studies improved the funnel plot symmetry, resulting in a pooled SAR of 26.0% (95% CI, 16.6%–38.3%).

### Quality Assessment

Using the modified Newcastle-Ottawa Quality Assessment Scale [2], we classified 9 studies as high quality, 14 as moderate quality, and 11 as low quality (Supplementary Table 2). Lower quality was mainly attributed to studies with 1 or an unspecified number of tests per contact (28 studies, 84.9%), not testing all identified contacts (17 studies, 51.5%), and not presenting SARs stratified by index case or contact characteristics (15 studies, 45.4%). The pooled SAR for the high-quality studies was 10.2% (95% CI, 3.3%–27.3%).

## DISCUSSION

Our systematic review provides evidence on the community indoor settings that have the highest risk of SARS-CoV-2 transmission. The SARs were the highest for singing events, community settings like indoor meetings and entertainment venues, and fitness centers. Rates were the lowest for workplaces. We found no difference in SARs by index case, viral, and setting-specific characteristics.

The exceptionally high SAR (44.9%) for singing events suggests that long-distance airborne transmission may have occurred in at least some events [8]. Singing may have increased the amount of virus-containing aerosols generated by the primary cases, which is consistent with experimental and modeling studies [55, 56]. Singing in poorly ventilated and overcrowded indoor spaces is particularly known to facilitate SARS-CoV-2 transmission [25, 31]. One study reported secondary transmission despite natural (open doors and windows) and mechanical ventilation at 2 choir venues in the Netherlands [46]. When considering risk mitigation in indoor settings, it is important to continue highlighting the risks of singing and loud vocalization, particularly in poorly ventilated spaces.

The risk of secondary transmission was high in fitness centers (28.9%). High-intensity exercise increases aerosol emission many fold compared to the resting state [57]. Many different high-intensity activities were reported in the fitness setting outbreaks [22, 34, 43, 48]. Other than exercise intensity, not wearing masks, extended close contact, and poor facility ventilation may also have facilitated SARS-CoV-2 transmission in specific settings [34, 43]. Two studies reported secondary transmission despite masking recommendations for nonactive participants [29, 43]. Only 1 study reported functioning ventilation systems during fitness classes [22]. Implementing universal mask wearing, reduced occupancy, improved facility ventilation, and virtual or outdoor exercise classes may reduce the risk of transmission in fitness centers [58, 59].

The pooled SAR (31.9%) for the other community settings, which did not have sufficient data to stratify further, was also high. The study-level SARs in this category were the highest for an indoor team meeting (40.0%) [38], a stay in a ski resort cabin (73.3%) [28], and a scientific meeting in a hotel (84.6%) [32]. Two of these outbreaks [28, 32] occurred early in the pandemic (January to February 2020) when no control measures were in place, and 1 occurred in the fall of 2020, even with participants sitting 1.8 meters (6 feet) apart and wearing cloth masks at all times [38]. Secondary transmission during the scientific meeting was hypothesized to have occurred due to aerosolization and extended face-to-face contact [32]. More data are needed to estimate summary SARs and the factors facilitating SARS-CoV-2 transmission in hotels, indoor meetings, and entertainment venues.

Dining settings are known to facilitate SARS-CoV-2 transmission due to the difficulty in wearing masks and maintaining social distancing during meals [60]. Nearly all the transmission events in this category occurred in restaurants [24, 27, 36, 47, 53] and 1 in shared eating areas in a university [37]. Outbreaks in restaurants occurred despite natural and mechanical ventilation [53], mechanical ventilation and air purification [27], and high COVID-19 vaccination coverage [24]. Revising ventilation standards, installing high-efficiency particulate air filters, and emphasizing masking and proper hygiene for staff handling food and beverages may help reduce SARS-CoV-2 spread in these settings [60, 61].

The setting-specific SAR (6.2%) was the lowest for workplaces. A previous meta-analysis reported a lower SAR (1.9%) for workplaces but highlighted the limited data available at the time [5]. Our estimate was based on 12 studies (15 transmission events) conducted in 7 countries in non-health care settings [26, 27, 37, 40, 42, 44, 45, 49, 50, 52, 54]. The low SARs at workplaces before the widespread availability of COVID-19 vaccines may have been due to timely control measures such as symptom monitoring and regularly testing employees, universal masking, social distancing, paid sick leave, and limiting capacity through worker bubbles and remote working [62, 63]. Many workplaces may also have implemented engineering controls like adding physical barriers between workers, improving airflow and ventilation, and enhancing cleaning practices [62]. The higher SAR for offices (compared with retail and unspecified workplaces) in our study could be associated with the closer proximity or longer duration of exposure to index cases in these settings, even though these data were not frequently reported. Future studies should investigate the impact of workplace policies on reducing SARS-CoV-2 transmission risks and the reasons for the relatively high SARs in offices.

We did not find any differences in SARs by study (study design, location, contact testing strategy, and duration of following contacts), index case (age and symptom status), viral (predominant circulating variant), and setting-specific (dimensions, exposure duration, ventilation, mask-wearing, and physical distancing measures) characteristics. Our subgroup analyses were limited when stratifying the SARs by setting-specific characteristics, which were not consistently reported in the included studies. Only 10 of 34 studies reported on ventilation, 15 on masking, and 12 on physical distancing recommendations in community indoor settings (Supplementary Table 4). Furthermore, there were differences in how these characteristics were reported in the included studies. The lack of standardized reporting makes investigating the factors that may modify SARS-CoV-2 transmission risks in community indoor settings challenging.

Our study has several limitations. First, we observed high heterogeneity between studies, possibly attributable to differences in index case and contact definitions, testing protocols, sociodemographic and setting characteristics, and local containment measures [3]. The overall and setting-specific SARs had a between-study heterogeneity (*I*^2^) of 86% or higher. We used random-effects models to estimate the SARs and conducted subgroup analyses on various study-level characteristics (particularly study design, location, and quality) to better account for and explain this heterogeneity. However, the lack of standardized reporting of study exposures also limited these analyses. The marked heterogeneity potentially limits conclusions drawn from our summary estimates. Second, the settings frequently appearing in our review may represent transmission events that were easier to detect and investigate because they involved defined groups with complete participant lists. Third, not all outbreaks in the settings of interest may have been published in peer-reviewed journals due to limited public health resources, prioritizing vaccine rollout, or COVID-19 containment activities [8]. Furthermore, contact tracing became more challenging in the fall of 2021 with the emergence of the Omicron variant, whose serial interval was significantly shorter than the earlier variants [64]. Finally, the pooled and setting-specific SARs were high (>18%), except for workplaces. Possible reasons for this may include biases in study design (failure to detect the existence of multiple index cases or not identifying all the contacts of index cases) [65] and publication bias (large outbreaks or those with higher super-spreading potential were more likely to be published). We did not find substantial asymmetry in our modified funnel plots, implying that inflated SARs due to publication bias were less likely.

In conclusion, although vaccines are the cornerstone of COVID-19 prevention, our study provides evidence of the added benefit of accounting for setting types and characteristics to strengthen prevention efforts for respiratory pathogens. Our findings suggest that the risk of SARS-CoV-2 transmission is highest in indoor settings where singing and exercising occur. Future studies should systematically assess and report the host (index case and contact characteristics), viral (variants and subvariants), and setting-specific (ventilation, masking, occupancy, and contact patterns) factors that may modify the transmission risks of SARS-CoV-2 and other respiratory viruses in indoor environments. We recommend tailored mitigation measures shown to be effective in high-risk indoor settings, such as assessing and improving ventilation.

## Supplementary Data

Supplementary materials are available at *The Journal of Infectious Diseases* online (http://jid.oxfordjournals.org/). Supplementary materials consist of data provided by the author that are published to benefit the reader. The posted materials are not copyedited. The contents of all supplementary data are the sole responsibility of the authors. Questions or messages regarding errors should be addressed to the author.

## Supplementary Material

jiae261_Supplementary_Data

## Notes

***Acknowledgments.*** The authors acknowledge the support of Päivi Lukin for developing the search strategy and implementing pilot searches in Ovid Medline and PubMed. We also acknowledge Drs Arto Säämänen, Ilpo Kulmala, Erja Mäkelä, Marika Lehtola, and Sirpa Laitinen for their insightful comments on the preliminary results of this systematic review.

***Author contributions.*** J. P. N. and M. R. F. conceptualized this study. M. R. F. worked with an information specialist to design the search strategy, conducted the database searches, and prepared the studies for screening. M. R. F. and S. G. performed the study screening, data extraction, and quality assessment. M. R. F. performed the analyses and wrote the first draft of the manuscript. J.P.N. obtained funding and supervised the study. All authors critically reviewed the manuscript and provided final approval prior to submission.

***Disclaimer.*** The funder had no role in study design, data collection and analysis, decision to publish, or preparation of the manuscript.

***Financial support.*** This work was supported by Business Finland “E3 Excellence in Pandemic Response and Enterprise Solutions” (grant number 4358/31/2021 to Tampere University).

***Data sharing.*** All the data collected from the included studies will be provided upon reasonable request. Requests should be directed to the corresponding author.
