# Supplementary material for: The Risk of SARS-CoV-2 Transmission in Community Indoor Settings: A Systematic Review and Meta-analysis
Source: J Infect Dis. 2024 May 16;230(4):e824–36. doi: 10.1093/infdis/jiae261 (PMC11481457; doi:10.1093/infdis/jiae261)
Supplement: jiae261_Supplementary_Data [file jiae261_supplementary_data.pdf]

Supplemental material to

The Risk of SARS-CoV-2 Transmission in Community Indoor Settings: A Systematic Review and Meta-Analysis

Mark Rohit Francis[1], Saheed Gidado[1], and J. Pekka Nuorti[1,2]

[1] Health Sciences Unit, Faculty of Social Sciences, Tampere University, Tampere, Finland

[2] Infectious Diseases and Vaccinations Unit, Department of Health Security, Finnish Institute for Health and Welfare (THL), Helsinki, Finland

Supplementary Appendix 1: Search strategy in MEDLINE via Ovid

Ovid MEDLINE(R) ALL <1946 to October 27, 2022>

1	exp SARS-CoV-2/ or SARS-CoV-2.tw.	174566
2	exp COVID-19/ or COVID-19.tw.	288395
3	exp Coronavirus/	153189
4	"SARS-CoV-2 variant*".tw.	3875
5	"Coronavirus variant*".tw.	107
6	"Covid-19 variant*".tw.	271
7	"severe acute respiratory syndrome*".tw.	35774
8	"sars cov*".tw.	92548
9	"SARS-CoV*".tw.	92548
10	Covid*.tw.	262756
11	coronavirus.tw.	104035
12	"corona virus".tw.	2744
13	covid-19.tw.	256985
14	"2019-nCoV".tw.	1538
15	"coronavirus disease".tw.	59853
16	Disease Transmission, Infectious/	10916
17	Disease Outbreaks/ or Disease Hotspot/ or "disease outbreak*".tw.	94785
18	exp Contact Tracing/ or "contact tracing".tw.	8375
19	"attack rate*".tw.	5200
20	"secondary attack rate*".tw.	506
21	"secondary transmission".tw.	592
22	"close contact*".tw.	11905
23	"index case*".tw.	7775
24	"indoor transmission".tw.	45
25	"contact transmission".tw.	518
26	superspread*.tw.	421
27	"super spread*".tw.	305
28	Workplace/	28408
29	Libraries/	2492

30 Restaurants/ 4330

31 Universities/ 50379

32 Museums/ 3847

33 Private Facilities/ 92

34 Public Facilities/1317

35 Fitness Centers/646

36 Call Centers/ 129

37 Faith-Based Organizations/ 145

38 (employee* or employer* or employed or worker* or workplace* or "work place*" or office* or universit* or college*).tw. 1313738

39 (meeting* or conference* or attendee* or exhibit* or fair* or camp* or course*).tw. 2341494

40 (restaurant* or cafe* or canteen* or cafeteria*).tw. 13019

41 (hotel* or hostel* or spa* or resort* or dorm*).tw. 1396914

42 (bar or bars or pub or pubs or club* or nightclub* or disco*).tw. 858905

43 (supermarket* or "super market*" or shop* or "department store*" or "departmental store*" or "grocery store*" or "indoor market*" or mall*).tw. 39033

44 (salon* or parlour* or parlor* or barber*).tw. 3756

45 ("gym" or "gyms" or "gymnasium" or "gymnasiums" or "sports event*" or "sporting event*" or "sports facilit*" or "sporting facilit*" or "sports center*" or "sporting center*" or stadium* or arena* or sauna* or bath*).tw. 83889

46 ("religious service*" or "religious ceremon*" or "religious gathering*" or worship* or church* or chapel* or cathedral* or mosque* or temple* or synagogue* or wedding* or pilgrim* or funeral*).tw. 18137

47 (concert* or choir* or party or parties or singing* or karaoke* or "movie theater*" or "movie theatre*" or cinema*).tw. 80490

48 (indoor* or hall* or building* or venue* or gathering* or "mass gathering*").tw. 320722

49 1 or 2 or 3 or 4 or 5 or 6 or 7 or 8 or 9 or 10 or 11 or 12 or 13 or 14 or 15 322695

50 16 or 17 or 18 or 19 or 20 or 21 or 22 or 23 or 24 or 25 or 26 or 27 131817

51 28 or 29 or 30 or 31 or 32 or 33 or 34 or 35 or 36 or 37 or 38 or 39 or 40 or 41 or 42 or 43 or 44 or 45 or 46 or 47 or 48 5829967

52 49 and 50 and 51 4742

53 limit 52 to (english language and yr="2020-Current") 4005

Supplementary Appendix 2: PRISMA 2020 checklist

Section and Topic	Item #	Checklist item	Location where item is reported
**TITLE**			
Title	1	Identify the report as a systematic review.	Title and lines 97-99
**ABSTRACT**			
Abstract	2	See the PRISMA 2020 for Abstracts checklist.	Lines 34-62
**INTRODUCTION**			
Rationale	3	Describe the rationale for the review in the context of existing knowledge.	Lines 70-97
Objectives	4	Provide an explicit statement of the objective(s) or question(s) the review addresses.	Lines 98-101
**METHODS**			
Eligibility criteria	5	Specify the inclusion and exclusion criteria for the review and how studies were grouped for the syntheses.	Lines 120-132 and 181-183
Information sources	6	Specify all databases, registers, websites, organisations, reference lists and other sources searched or consulted to identify studies. Specify the date when each source was last searched or consulted.	Lines 105-117
Search strategy	7	Present the full search strategies for all databases, registers and websites, including any filters and limits used.	Supplementary Appendix 1
Selection process	8	Specify the methods used to decide whether a study met the inclusion criteria of the review, including how many reviewers screened each record and each report retrieved, whether they worked independently, and if applicable, details of automation tools used in the process.	Lines 135-139
Data collection process	9	Specify the methods used to collect data from reports, including how many reviewers collected data from each report, whether they worked independently, any processes for obtaining or confirming data from study investigators, and if applicable, details of automation tools used in the process.	Lines 140-148
Data items	10a	List and define all outcomes for which data were sought. Specify whether all results that were compatible with each outcome domain in each study were sought (e.g. for all measures, time points, analyses), and if not, the methods used to decide which results to collect.	Lines 150-157
	10b	List and define all other variables for which data were sought (e.g. participant and intervention characteristics, funding sources). Describe any assumptions made about any missing or unclear information.	Lines 150-157
Study risk of bias assessment	11	Specify the methods used to assess risk of bias in the included studies, including details of the tool(s) used, how many reviewers assessed each study and whether they worked independently, and if applicable, details of automation tools used in the process.	Lines 160-170
Effect measures	12	Specify for each outcome the effect measure(s) (e.g. risk ratio, mean difference) used in the synthesis or presentation of results.	Lines 155-157 and 172-175
Synthesis methods	13a	Describe the processes used to decide which studies were eligible for each synthesis (e.g. tabulating the study intervention characteristics and comparing against the planned groups for each synthesis (item #5)).	Line 172

Section and Topic	Item #	Checklist item	Location where item is reported
	13b	Describe any methods required to prepare the data for presentation or synthesis, such as handling of missing summary statistics, or data conversions.	Lines 175-178
	13c	Describe any methods used to tabulate or visually display results of individual studies and syntheses.	Lines 195-199
	13d	Describe any methods used to synthesize results and provide a rationale for the choice(s). If meta-analysis was performed, describe the model(s), method(s) to identify the presence and extent of statistical heterogeneity, and software package(s) used.	Lines 173-180 and 197-199
	13e	Describe any methods used to explore possible causes of heterogeneity among study results (e.g. subgroup analysis, meta-regression).	Lines 181-188
	13f	Describe any sensitivity analyses conducted to assess robustness of the synthesized results.	Lines 191-195
Reporting bias assessment	14	Describe any methods used to assess risk of bias due to missing results in a synthesis (arising from reporting biases).	Lines 194-197
Certainty assessment	15	Describe any methods used to assess certainty (or confidence) in the body of evidence for an outcome.	N/A
**RESULTS**			
Study selection	16a	Describe the results of the search and selection process, from the number of records identified in the search to the number of studies included in the review, ideally using a flow diagram.	Lines 203-208 and Figure 1
	16b	Cite studies that might appear to meet the inclusion criteria, but which were excluded, and explain why they were excluded.	N/A
Study characteristics	17	Cite each included study and present its characteristics.	Table 1 and lines 211-226
Risk of bias in studies	18	Present assessments of risk of bias for each included study.	Supplementary Table 2
Results of individual studies	19	For all outcomes, present, for each study: (a) summary statistics for each group (where appropriate) and (b) an effect estimate and its precision (e.g. confidence/credible interval), ideally using structured tables or plots.	N/A
Results of syntheses	20a	For each synthesis, briefly summarise the characteristics and risk of bias among contributing studies.	Lines 230-254 and 272-278
	20b	Present results of all statistical syntheses conducted. If meta-analysis was done, present for each the summary estimate and its precision (e.g. confidence/credible interval) and measures of statistical heterogeneity. If comparing groups, describe the direction of the effect.	Figure 2
	20c	Present results of all investigations of possible causes of heterogeneity among study results.	Lines 255-260
	20d	Present results of all sensitivity analyses conducted to assess the robustness of the synthesized results.	Lines 262-270
Reporting biases	21	Present assessments of risk of bias due to missing results (arising from reporting biases) for each synthesis assessed.	Lines 266-270
Certainty of evidence	22	Present assessments of certainty (or confidence) in the body of evidence for each outcome assessed.	N/A
**DISCUSSION**			

Section and Topic	Item #	Checklist item	Location where item is reported
Discussion	23a	Provide a general interpretation of the results in the context of other evidence.	Lines 286-336
	23b	Discuss any limitations of the evidence included in the review.	Lines 348-368
	23c	Discuss any limitations of the review processes used.	N/A
	23d	Discuss implications of the results for practice, policy, and future research.	Lines 369-377
**OTHER INFORMATION**			
Registration and protocol	24a	Provide registration information for the review, including register name and registration number, or state that the review was not registered.	Lines 114-116
	24b	Indicate where the review protocol can be accessed, or state that a protocol was not prepared.	Lines 114-116
	24c	Describe and explain any amendments to information provided at registration or in the protocol.	Lines 183-186
Support	25	Describe sources of financial or non-financial support for the review, and the role of the funders or sponsors in the review.	Lines 392-396
Competing interests	26	Declare any competing interests of review authors.	Lines 398-401
Availability of data, code and other materials	27	Report which of the following are publicly available and where they can be found: template data collection forms; data extracted from included studies; data used for all analyses; analytic code; any other materials used in the review.	All study material are available upon request to the corresponding author

Supplementary Table 1: Index case and contact definitions as reported in the included studies

Main text reference	Author	Study setting	Index case definition	Contact definition
22	Bae et al. (2020) [1]	Fitness centers	Index case was the first reported case	Any person who had been within 2 m from a confirmed case from 1 day before symptom onset of the case, without appropriate personal protection
23	Bao et al. (2021) [2]	Bathing pool	Index case classified by date of symptom onset/or test dates	All customers and workers at the bathing pool during the exposure period were considered contacts
24	Brandal et al. (2021) [3]	Restaurant	Index case was the first reported case	All attendees at the party on 26 November 2021
25	Charlotte et al. (2023) [4]	Indoor choir practice	Index case classified by date of symptom onset/or test dates	Unclear
26	Chaw et al. (2020) [5]	Religious gathering	Index case classified by date of symptom onset/or test dates	Any person who had been within 1m of a confirmed case-patient in an enclosed space for >15 minutes
		Workplaces	Same as above	Same as above
27	Cheng et al. (2022) [6]	Restaurant staff	Index case classified by date of symptom onset/or test dates	All who were exposed to the index case on Dec 27, 2021 during 13:00-15:00 in the restaurant
		Restaurant customers	Same as above	Same as above
28	Danis et al. (2020) [7]	Chalet	Index case classified by date of symptom onset/or test dates	Contacts were defined based on the level of risk of infection (negligible, low, and moderate risk), the time of exposure, proximity, and setting of exposure

29	Dougherty et al. (2021) [8]	Gymnastics facility	Index case classified by date of symptom onset/or test dates	Gymnast cohorts and staff members identified as attending facility A during April 15-May 3, 2021
30	Groves et al. (2021) [9]	Fitness center	Index case classified by date of symptom onset/or test dates	Fitness class participants and facility staff members considered close contacts
31	Hamner et al. (2020) [10]	Choir	Index case classified by date of symptom onset/or test dates	Probable cases were persons who attended the March 10 practice and developed clinically compatible COVID-19 symptoms as defined by council of state and territorial epidemiologists
32	Hijnen et al. (2020) [11]	Hotel meeting room	Index case classified by date of symptom onset/or test dates	Participants in contact with the index case during the scientific advisory board meeting held on Feb 20-21, 2020
33	James et al. (2020) [12]	Church	Index case was the first reported case	Persons who attended church A events during March 6-11, 2020
34	Jang et al. (2020) [13]	Fitness facility	Index case classified by date of symptom onset/or test dates	Unclear
35	Katelaris et al. (2021) [14]	Church	Index case classified by date of symptom onset/or test dates	Anyone who had spent >15 min face-to-face or shared a closed space for 2 hours with a case-patient during the infectious period of the case-patient
36	Lam et al. (2021) [15]	Restaurant	Index case classified by date of symptom onset/or test dates	All the relatives who attended the Chinese new year dinner on 26 Jan 2020
37	Montecucco et al. (2021) [16]	Offices	Not specified	Any person who had exposure to a probable or confirmed case or had direct and face-to-face contact exposure with the index case in the period between two days before the positive PCR test or two days preceding the onset of COVID-19 symptoms and end of isolation after infection resolution

		Shared eating areas	Not specified	Same as above
		Classrooms	Not specified	Same as above
38	Moreno et al. (2021) [17]	Indoor meeting	Index case classified by date of symptom onset/or test dates	Student athletes and staff who had close contact with the index case during the team meeting
39	Muller et al. (2021) [18]	Nightclub	Index case classified by date of symptom onset/or test dates	Attendees of events held at nightclub X between Feb 29 and March 5 - Those who attended 1 or more events were categorized as high-risk contacts
40	Ng et al. (2021) [19]	Workplaces	Index case classified by date of symptom onset/or test dates	Individuals who came into close contact with the index case at work, from 2 days before the onset of symptoms to isolation of the case, to account for pre-symptomatic transmission
-	Noman et al. (Preprint) [20]	Funeral	Index case classified by date of symptom onset/or test dates	Close contacts were individuals exposed to index cases in households, funerals, and other settings
		Workplace	Same as above	Same as above
41	Nsekuye et al. (2021) [21]	Nightclub	Index case classified by date of symptom onset/or test dates	Any person who had contact with a COVID-19 case within a timeframe ranging from 72h before onset of symptoms for the case to 14 d after the onset of symptoms
42	Park et al. (2020) [22]	Office	Index case classified by date of symptom onset/or test dates	Persons who worked at, lived at, or visited Building X during February 21 - March 8, 2020
		Call center office	Same as above	Same as above

43	Pauser et al. (2021) [23]	Sporting facility	Index case classified by date of symptom onset/or test dates	All players, coaches and other persons present at the sporting event were investigated as potential contacts
44	Sarti et al. (2021) [24]	Office	Index case classified by date of symptom onset/or test dates	Unclear
45	Seok et al. (2022) [25]	Workplaces	Index case classified by date of symptom onset/or test dates	Contacts of all confirmed COVID-19 cases were defined based on route of movement for 2 days before symptom onset (proxy for the degree of contact)
		Religious gatherings	Same as above	Same as above
		Gyms and fitness centers	Same as above	Same as above
46	Shah et al. (Preprint) [26]	Singing Event 4	Index case classified by date of symptom onset/or test dates	Unclear
		Singing Event 5	Same as above	Unclear
		Singing Event 3	Same as above	Unclear
47	Shen et al. (2020) [27]	Lunch	Index case was the first reported case	Close contacts were individuals who had close, prolonged, and repeated interactions with the two source cases
48	Shin et al. (2022) [28]	Taekwondo gym	Index case classified by date of symptom onset/or test dates	Close contacts were classified based on an evaluation of risk levels such as exposure range (time, place, etc.) with confirmed patients and whether or not a person wore a mask

49	Sundar et al. (2021) [29]	Indoor workplaces	Index case classified by date of symptom onset/or test dates	Contacts were those exposed to the index case in the pre-symptomatic (2 days prior to symptom onset) or symptomatic period and persons at workplaces who were exposed to the index case at close range (less than 6 feet) for ≥15 minutes
50	Tian et al. (2021) [30]	Supermarket employees	Index case classified by date of symptom onset/or test dates	All the supermarket employees who had contact with the index case and people who visited the supermarket from January 15 to January 22, 2020
51	Yusef et al. (2020) [31]	Wedding	Index case classified by date of symptom onset/or test dates	Persons who attended the wedding or had close contact with attendees
52	Zhang et al. (2020) [32]	Workplaces	Index case classified by date of symptom onset/or test dates	Close contacts were persons who had contact with an asymptomatic index case without using proper protection during 2 days before the index case was tested
53	Zhang et al. (2022) [33]	Restaurant	Not specified	Unclear
54	Zhou et al. (2022) [34]	Salespersons	Index case classified by date of symptom onset/or test dates	Individuals who have been exposed to a confirmed COVID-19 case within 2 days prior to the onset of symptoms
		Administrators	Same as above	Same as above

Supplementary Table 2: Quality assessment of the included studies using the Modified Newcastle-Ottawa scale [35]

		Scale items						Study rating	
**Main text reference**	**Author (Publication year)**	**Index case definition (1 point)[a]**	**Sample size (1 point)[b]**	**SAR stratified by index and/or contact characteristics (1 point)[c]**	**Contact testing strategy (1 point)[d]**	**Follow-up duration of negative contacts (2 points)[e]**	**Number of tests per contact (1 point)[f]**	**Total score (max. 7 points)**	**Quality[g]**
22	Bae et al. (2020) [1]	0	+	+	0	+	0	3	Moderate
23	Bao et al. (2021) [2]	+	0	+	0	+	0	3	Moderate
24	Brandal et al. (2021) [3]	0	+	+	+	++	0	5	High
25	Charlotte et al. (2023) [4]	+	0	+	0	0	0	2	Low
26	Chaw et al. (2020) [5]	+	+	+	+	+	0	5	High
27	Cheng et al. (2022) [6]	+	+	+	+	0	0	4	Moderate
28	Danis et al. (2020) [7]	+	0	0	0	+	0	2	Low
29	Dougherty et al. (2021) [8]	+	0	+	0	0	0	2	Low

30	Groves et al. (2021) [9]	+	0	+	0	+	0	3	Moderate
31	Hamner et al. (2020) [10]	+	0	+	0	+	0	3	Moderate
32	Hijnen et al. (2020) [11]	+	0	0	0	0	0	1	Low
33	James et al. (2020) [12]	0	+	+	0	+	0	3	Moderate
34	Jang et al. (2020) [13]	+	+	0	+	0	0	3	Moderate
35	Katelaris et al. (2021) [14]	+	+	0	+	++	+	6	High
36	Lam et al. (2021) [15]	+	0	0	0	0	0	1	Low
37	Montecucco et al. (2021) [16]	0	+	+	0	0	0	2	Low
38	Moreno et al. (2021) [17]	+	0	0	+	+	+	4	Moderate
39	Muller et al. (2021) [18]	+	+	0	0	0	0	2	Low
40	Ng et al. (2021) [19]	+	+	+	0	+	0	4	Moderate

-	Noman et al. (Preprint) [20]	+	+	+	+	+	0	5	High
41	Nsekuye et al. (2021) [21]	+	+	+	+	+	0	5	High
42	Park et al. (2020) [22]	+	+	0	+	+	+	5	High
43	Pauser et al. (2021) [23]	+	0	0	+	0	0	2	Low
44	Sarti et al. (2021) [24]	+	0	+	+	+	0	4	Moderate
45	Seok et al. (2022) [25]	+	+	+	+	0	0	4	Moderate
46	Shah et al. (Preprint) [26]	+	0	0	0	+	0	2	Low
47	Shen et al. (2020) [27]	0	0	0	0	1	0	1	Low
48	Shin et al. (2022) [28]	+	+	+	+	+	0	5	High
49	Sundar et al. (2021) [29]	+	+	0	+	0	0	3	Moderate

50	Tian et al. (2021) [30]	+	+	0	+	0	+	4	Moderate
51	Yusef et al. (2020) [31]	+	+	0	+	++	0	5	High
52	Zhang et al. (2020) [32]	+	+	+	0	+	+	5	High
53	Zhang et al. (2022) [33]	0	0	0	+	0	0	1	Low
54	Zhou et al. (2022) [34]	+	+	+	0	+	0	4	Moderate

[a] +: By date of symptom onset and/or test dates; 0: First reported case or not specified

[b] +: 70 or more contacts; 0: Less than 70 contacts

[c] +: Secondary attack rate stratified by one or more covariates; 0: Secondary attack rate not stratified by any covariates

[d] +: Tested all contacts regardless of symptoms; 0: Only tested symptomatic contacts

[e] ++: > 14 days; +: 14 days; 0: Fewer than 14 days or not specified

[f] +: Two or more tests; 0: One test or not described

[g] Low quality: 1-2 points: Moderate quality: 3-4 points; High quality: 5-7 points

Supplementary Table 3: Reasons for excluding studies after full-text review

Reason for exclusion	Databases		Citation searches	
	n	%	*n*	%
No secondary transmission reported	2	7.1	1	16.7
Unclear if secondary transmission occurred	6	21.4	1	16.7
Index case unidentified	8	28.6	1	16.7
Contacts not applicable	1	3.6	0	0.0
Unclear or excluded setting	7	25.0	2	33.3
Total contacts unknown	3	10.7	1	16.7
Data not original	1	3.6	0	0.0
Duplicate study	2	7.1	0	0.0

Supplementary Table 4: The setting-specific factors potentially influencing the risk of SARS-CoV-2 transmission in community indoor setting in the included studies, January 1, 2020 – February 20, 2023 (n = 34 studies, 45 transmission events)

Main text reference	Author (Year published)	Study setting	Exposure duration	Proximity of index cases/contacts	Ventilation		Masking			Physical distancing		
					Type	Specification	Recommendation	Adherence*	Specification	Recommendation	Adherence*	Specification
22	Bae et al. (2020) [1]	Fitness centers	Not specified	Contacts were persons within 2 meters of index cases	Not specified	Ventilation systems were reportedly working well	Not specified	Not specified	Not specified	No	Not specified	Physical distancing measures initiated after the outbreak
23	Bao et al. (2021) [2]	Other setting	2.5 hours per day (total not specified)	Not specified	Not specified	Not specified	Not specified	Not specified	Not specified	Not specified	Not specified	Not specified
24	Brandal et al. (2021) [3]	Dining setting	4.5 hours	Not specified	Not specified	Not specified	No	Not specified	Not specified	Not specified	Not specified	Not specified
25	Charlotte et al. (2023) [4]	Singing event	2 hours	Not specified	None	The room was not ventilated	No	Not specified	Not specified	Yes	Not specified	Choir members sat a little less than 6 feet apart
26	Chaw et al. (2020) [5]	Religious setting	Not specified	Contacts were persons within 1 meter of a confirmed case	Not specified	Not specified	Not specified	Not specified	Not specified	Not specified	Not specified	Moderate physical distancing was enforced before cluster identification
		Workplaces	Not specified		Not specified	Not specified	Not specified	Not specified	Not specified	Not specified	Not specified	

27	Cheng et al. (2022) [6]	Workplace	2 hours	Not specified	Mechanical	Ultraviolet-C air purifiers were operational when the index case visited	Yes	Not specified	Restaurant staff were required to wear masks	Not specified	Not specified	Not specified
		Dining setting	2 hours	Not specified	Mechanical		No	Not specified	Customers did not wear masks while in the dining area	Not specified	Not specified	Not specified
28	Danis et al. (2020) [7]	Other setting	Not specified	Proximity between cases/contacts considered but distance not specified	Not specified	Not specified	Not specified	Not specified	Not specified	Not specified	Not specified	Not specified
29	Dougherty et al. (2021) [8]	Fitness center	Not specified	Not specified	Not specified	Poor facility ventilation reported	No	Not specified	Mask wearing prevalence was low among those not participating	Not specified	Not specified	Not specified
30	Groves et al. (2021) [9]	Fitness center	1-hour sessions (total not specified)	Not specified	None	Doors and windows were closed/poor facility ventilation reported	No	Not specified	Use of masks was not required in fitness facilities	Yes	Yes, but not specified	Instructor and participants were more than 6 feet apart
31	Hamner et al. (2020) [10]	Singing event	2.5 hours	Not specified	Not specified	Not specified	Not specified	Not specified	Not specified	No	Not specified	Transmission likely facilitated by proximity during practice

32	Hijnen et al. (2020) [11]	Other setting	9.5 hours	Not specified	Not specified	No data about room ventilation and airflow patterns	Not specified	0%	No masks were worn during the meeting	Not specified	Not specified	Not specified
33	James et al. (2020) [12]	Religious setting	5 hours across three days	Not specified	Not specified	Not specified	Not specified	Not specified	Not specified	Not specified	Not specified	Not specified
34	Jang et al. (2020) [13]	Fitness centers	50 minutes per class	Not specified	Not specified	Not specified	Not specified	Not specified	Not specified	Not specified	Not specified	Not specified
35	Katelaris et al. (2021) [14]	Singing event	4 x 1-hour services	Index case was around 15 meters from the contacts	None	Ventilation systems were not operational/Doors and windows were closed during services	No	Not specified	Not specified	Yes	Not specified	There was a 3-meter cordon between the singers and congregation
36	Lam et al. (2021) [15]	Dining setting	About 7 hours	Not specified	Not specified	Not specified	Not specified	Not specified	Not specified	Not specified	Not specified	Close contact activities including dining and mahjong were done
37	Montecucco et al. (2021) [16]	Workplace	Not specified	Not specified	Not specified	Not specified	Not specified	66% of the index cases/contacts across settings reported wearing surgical	Mask wearing appeared mandatory in offices and classrooms, but implementation may have	Not specified	Not specified	Not specified
		Dining setting	Not specified	Not specified	Not specified	Not specified	Not specified			Not specified	Not specified	Not specified

		Workplace	Not specified	Not specified	Not specified	Not specified	Not specified	masks or filtering facepieces	been less feasible in other settings	Not specified	Not specified	Not specified
38	Moreno et al. (2021) [17]	Other setting	Not specified	Index case and contacts were reportedly 6 feet apart	Not specified	Not specified	Yes	100 %	All athletes always wore cloth masks during the indoor meeting	Yes	100 %	All students and staff were to be at least 6 feet apart during meetings
39	Muller et al. (2021) [18]	Other setting	Not specified	Not specified	Not specified	Not specified	Not specified	Not specified	Not specified	No	Not specified	The outbreak occurred in the early phase of the pandemic before prevention measures were applied
40	Ng et al. (2021) [19]	Workplaces	Not specified	Proximity between cases/contacts considered but distance not specified	Not specified	Not specified	Not specified	Low	Prevalence of mask use was low at that time	Not specified	Not specified	Not specified
-	Noman et al. (Preprint) [20]	Religious setting	Not specified	Not specified	Not specified	Not specified	Not specified	Not specified	Not specified	Not specified	Not specified	Not specified
		Workplaces	Not specified	Not specified	Not specified	Not specified	Not specified	Not specified	Not specified	Not specified	Not specified	Not specified

41	Nsekuye et al. (2021) [21]	Other setting	Not specified	Contacts were classified based on face-to-face contact within 2 meters	Not specified	Not specified	Not specified	Not specified	Not specified	Not specified	Not specified	Not specified
42	Park et al. (2020) [22]	Workplace	Not specified	Not specified	Not specified	Not specified	Not specified	Not specified	Not specified	Not specified	Not specified	Not specified
		Workplace	Not specified	Not specified	Not specified	Not specified	Not specified	Not specified	Not specified	Not specified	Not specified	Not specified
43	Pauser et al. (2021) [23]	Fitness centers	Not specified	Not specified	Not specified	Data on exchange rates of air an air flow were not collected	Not specified	47 %	The masks were either medical or particle filter face coverings	Yes	Not specified	Physical distancing recommended for sporting staff other than athletes (1.5 meters apart)
44	Sarti et al. (2021) [24]	Workplace	8 hours per day (total not specified)	Distance between index cases and contacts was between 1.8 to 5 meters	Not specified	The office had two large windows, but was considered high risk since air ventilation was poor	Yes	Low	No face covering was worn for a prolonged period even though required by the employers	Yes	Not specified	Coworkers were at least 1 meter apart from each other
45	Seok et al. (2022) [25]	Workplaces	Not specified	Not specified	Not specified	Not specified	Not specified	Not specified	Wearing of face masks was not assessed	Not specified	Not specified	Social distancing was not monitored

		Religious settings	Not specified	Not specified	Not specified	Not specified	Not specified	Not specified		Not specified	Not specified	
		Fitness centers	Not specified	Not specified	Not specified	Not specified	Not specified	Not specified		Not specified	Not specified	
46	Shah et al. (Preprint) [26]	Singing event	2.5 hours	Most participants kept 1.5 meters distance during the rehearsals/performances	Mechanical and natural	Open doors and windows reported along with ceiling ventilation	No	Not specified	Face mask use in indoor places was not obligatory during the study period	Not specified	>95%	Most participants kept 1.5 meters distance during the rehearsals/performances
		Singing event	2 hours		Mechanical and natural	Open doors and windows reported along with possible mechanical ventilation	No	Not specified		Not specified	100 %	
		Singing event	1 hour		Natural	Open doors and windows during the event	No	Not specified		Not specified	100 %	
47	Shen et al. (2020) [27]	Dining setting	Not specified	Proximity between cases/contacts considered but distance not specified	Not specified	Not specified	Not specified	Not specified	Not specified	Not specified	Not specified	Not specified
48	Shin et al. (2022) [28]	Fitness center	Not specified	Not specified	Not specified	Windows were closed during classes and ventilation was conducted between sessions	Yes	79 %	Students and staff reported always wearing masks during classes	Not specified	Not specified	Not specified

49	Sundar et al. (2021) [29]	Workplace	Average duration of exposure between was 44 minutes	Contacts were persons who were exposed to index cases at close range (< 6 feet)	Not specified	Information on ventilation was not collected	Yes	28 %	Mask wearing was required but adherence in indoor workspaces was low	Not specified	22 %	Contacts who maintained 6 feet or more with index cases were adherent
50	Tian et al. (2021) [30]	Workplaces	Not specified	Not specified	Not specified	Not specified	Not specified	Not specified	Not specified	Not specified	Not specified	Not specified
51	Yusef et al. (2020) [31]	Religious setting	2 hours	Not specified	Not specified	Not specified	Not specified	Not specified	Not specified	Not specified	Not specified	Not specified
52	Zhang et al. (2020) [32]	Workplaces	Not specified	Not specified	Not specified	Not specified	Not specified	Not specified	Not specified	Yes	Not specified	Social distancing was enforced in the region
53	Zhang et al. (2022) [33]	Dining setting	About 2 hours	Not specified	Mechanical	Indoor ventilation rate considered adequate (> 5 air changes per hour)	Not specified	Not specified	Not specified	Not specified	Not specified	Not specified
54	Zhou et al. (2022) [34]	Workplace	Not specified	Not specified	Not specified	Ventilation of the building was rated poor before the outbreak	Not specified	Not specified	Not specified	Not specified	Not specified	Not specified
		Workplace	Not specified	Not specified	Not specified		Not specified	Not specified	Not specified	Not specified	Not specified	Not specified

* Adherence to masking or physical distancing was reported as the proportion of occupants wearing masks or complying with distancing recommendations in each setting.

Supplementary Appendix 3: Description of the setting-specific factors potentially associated with the risk of SARS-CoV-2 transmission.

Nine studies reported the duration of exposure of contacts with index cases, ranging from 1 hour [26] to 9.5 hours [11] (Supplementary Table 4). The proximity of contacts and index cases was reported in four studies [14,17,24,26]. The distance between index cases and contacts during transmission events ranged from around 1.5 meters [26] to 15 meters [14]. Ten studies [1,4,6,8,9,14,24,26,33,34] provided some information on ventilation in the indoor settings. A study [26] reported natural ventilation (open windows and/or doors) at two singing events, and three studies [6,26,33] reported functioning ventilation systems. Ventilation was not available or rated poor by the investigators in six studies [4,8,9,14,24,34]. Fifteen studies [1,3,4,6,8,9,11,14,16,17,19,23,24,26,29] reported about mask use in the included settings (Supplementary Table 4). Nine studies [8,11,16,17,19,23,24,28,29] provided information on masking adherence (the proportion of individuals wearing masks in specific settings/events), ranging from 0% [11] to 100% [17]. Three of these studies [8,19,24] did not provide adherence proportions but reported that the prevalence of mask use was low during the study period. Five studies [3,4,9,14,26] reported that mask-wearing was not obligatory in the investigated settings/events, and one study [6] reported a recommendation for masking for restaurant staff but not customers. The type of masks (cloth, surgical, or particle-filtering masks) were reported in three studies [16,17,23]. Twelve studies [1,4,9,10,14,17,18,23,24,26,29,32] provided physical distancing information during the investigations. Eight studies [4,9,14,17,23,24,26,32] reported regional or setting-specific physical distancing recommendations, and most recommendations were for 6 feet or more between occupants.

Supplementary references

1. Bae S, Kim H, Jung TY, et al. Epidemiological Characteristics of COVID-19 Outbreak at Fitness Centers in Cheonan, Korea. J Korean Med Sci **2020**; 35:e288.

2. Bao C, Pan E, Ai J, et al. COVID-19 outbreak following a single patient exposure at an entertainment site: An epidemiological study. Transbound Emerg Dis **2021**; 68:773–781.

3. Brandal L, MacDonald E, Veneti L, et al. Outbreak caused by the SARS-CoV-2 Omicron variant in Norway, November to December 2021. Euro Surveill **2021**; 26:2101147.

4. Charlotte N. High Rate of SARS-CoV-2 Transmission Due to Choir Practice in France at the Beginning of the COVID-19 Pandemic. J Voice **2023**; 37:292.e9-292.e14.

5. Chaw L, Koh WC, Jamaludin SA, Naing L, Alikhan MF, Wong J. SARS-CoV-2 transmission in different settings: Analysis of cases and close contacts from the Tablighi cluster in Brunei Darussalam. **2020**; Available at: https://www.medrxiv.org/content/10.1101/2020.05.04.20090043v2?versioned=TRUE.

6. Cheng VC-C, Lung DC, Wong S-C, et al. Outbreak investigation of airborne transmission of Omicron (B.1.1.529) - SARS-CoV-2 variant of concern in a restaurant: Implication for enhancement of indoor air dilution. J Hazard Mater **2022**; 430:128504–128504.

7. Danis K, Epaulard O, Benet T, et al. Cluster of Coronavirus Disease 2019 (COVID-19) in the French Alps, February 2020. Clin Infect Dis **2020**; 71:825–832.

8. Dougherty K, Mannell M, Naqvi O, Matson D, Stone J. SARS-CoV-2 B.1.617.2 (Delta) Variant COVID-19 Outbreak Associated with a Gymnastics Facility - Oklahoma, April-May 2021. MMWR Morb Mortal Wkly Rep **2021**; 70:1004–1007.

9. Groves LM, Usagawa L, Elm J, et al. Community Transmission of SARS-CoV-2 at Three Fitness Facilities - Hawaii, June-July 2020. MMWR Morb Mortal Wkly Rep **2021**; 70:316–320.

10. Hamner L, Dubbel P, Capron I, et al. High SARS-CoV-2 Attack Rate Following Exposure at a Choir Practice - Skagit County, Washington, March 2020. MMWR Morb Mortal Wkly Rep **2020**; 69:606–610.

11. Hijnen D, Marzano AV, Eyerich K, et al. SARS-CoV-2 Transmission from Presymptomatic Meeting Attendee, Germany. Emerg Infect Dis **2020**; 26:1935–1937.

12. James A, Eagle L, Phillips C, et al. High COVID-19 Attack Rate Among Attendees at Events at a Church - Arkansas, March 2020. MMWR Morb Mortal Wkly Rep **2020**; 69:632–635.

13. Jang S, Han SH, Rhee J-Y. Cluster of Coronavirus Disease Associated with Fitness Dance Classes, South Korea. Emerg Infect Dis **2020**; 26:1917–1920.

14. Katelaris AL, Wells J, Clark P, et al. Epidemiologic Evidence for Airborne Transmission of SARS-CoV-2 during Church Singing, Australia, 2020. Emerg Infect Dis **2021**; 27:1677–1680.

15. Lam T-S, Wong C-H, Lam W-H, et al. Coronavirus disease 2019 (COVID-19) outbreak during a Chinese New Year dinner in a restaurant, Hong Kong Special Administrative Region SAR (China), 2020. Western Pac Surveill Response J **2021**; 12:32–34.

16. Montecucco A, Dini G, Rahmani A, et al. Investigating SARS-CoV-2 transmission among co-workers in a University of Northern Italy during COVID-19 pandemic: an observational study. Med Lav **2021**; 112:429–435.

17. Moreno GK, Braun KM, Pray IW, et al. Severe Acute Respiratory Syndrome Coronavirus 2 Transmission in Intercollegiate Athletics Not Fully Mitigated With Daily Antigen Testing. Clin Infect Dis **2021**; 73:S45–S53.

18. Muller N, Kunze M, Steitz F, et al. Severe Acute Respiratory Syndrome Coronavirus 2 Outbreak Related to a Nightclub, Germany, 2020. Emerg Infect Dis **2020**; 27:645–648.

19. Ng OT, Marimuthu K, Koh V, et al. SARS-CoV-2 seroprevalence and transmission risk factors among high-risk close contacts: a retrospective cohort study. Lancet Infect Dis **2021**; 21:333–343.

20. Noman AS Karim, Mohammed Rezaul, Zahed, ASM, Karim ATMR Islam, Syed. Epidemiology and contact tracing assessment of COVID-19 and potential risk of transmission at different exposure settings: A prospective cohort study. **2021**; Available at: https://doi.org/10.21203/rs.3.rs-442709/v1.

21. Nsekuye O, Rwagasore E, Muhimpundu M, et al. Investigation of Four Clusters of Severe Acute Respiratory Syndrome Coronavirus 2 (SARS-CoV-2) in Rwanda, 2020. Int J Environ Res Public Health **2021**; 18:7018.

22. Park SY, Kim Y-M, Yi S, et al. Coronavirus Disease Outbreak in Call Center, South Korea. Emerg Infect Dis **2020**; 26:1666–1670.

23. Pauser J, Schwarz C, Morgan J, Jantsch J, Brem M. SARS-CoV-2 transmission during an indoor professional sporting event. Sci Rep **2021**; 11:20723.

24. Sarti D, Campanelli T, Rondina T, Gasperini B. COVID-19 in Workplaces: Secondary Transmission. Ann Work Expo Health **2021**; 65:1145–1151.

25. Seok H, Lim S, Kim J, et al. Infectivity of Coronavirus Disease 2019: A Prospective Cohort Study in the Korean Metropolitan Area. J Korean Med Sci **2022**; 37:e106.

26. Shah AA, Dusseldorp F, Veldhuijzen IK, et al. High SARS-CoV-2 attack rates following exposure during five singing events in the Netherlands, September-October 2020. medRxiv **2021**; Available at: https://www.medrxiv.org/content/10.1101/2021.03.30.21253126v2?versioned=TRUE.

27. Shen Y, Xu W, Li C, et al. A Cluster of Novel Coronavirus Disease 2019 Infections Indicating Person-to-Person Transmission Among Casual Contacts From Social Gatherings: An Outbreak Case-Contact Investigation. Open Forum Infect Dis **2020**; 7:ofaa231.

28. Shin SH, Park E, Kim S, et al. COVID-19 outbreak and risk factors for infection in a taekwondo gym in the Republic of Korea. Osong Public Health Res Perspect **2022**; 13:162–170.

29. Sundar V, Bhaskar E. Low secondary transmission rates of SARS-CoV-2 infection among contacts of construction laborers at open air environment. Germs **2021**; 11:128–131.

30. Tian S, Wu M, Chang Z, et al. Epidemiological investigation and intergenerational clinical characteristics of 24 coronavirus disease patients associated with a supermarket cluster: a retrospective study. BMC Public Health **2021**; 21:647.

31. Yusef D, Hayajneh W, Awad S, et al. Large Outbreak of Coronavirus Disease among Wedding Attendees, Jordan. Emerg Infect Dis **2020**; 26.

32. Zhang W, Cheng W, Luo L, et al. Secondary Transmission of Coronavirus Disease from Presymptomatic Persons, China. Emerg Infect Dis **2020**; 26:1924–1926.

33. Zhang N, Hu T, Zhang J, et al. Probable close contact transmission in a restaurant in China. J Infect **2022**; 85:573–607.

34. Zhou Y, Xiang F, Ni C, et al. Travel-associated outbreak of COVID-19 in a departmental store, Wenzhou, China. J Infect Dev Ctries **2022**; 16:752–758.

35. Fung HF, Martinez L, Alarid-Escudero F, et al. The Household Secondary Attack Rate of Severe Acute Respiratory Syndrome Coronavirus 2 (SARS-CoV-2): A Rapid Review. Clin Infect Dis **2021**; 73:S138–S145.
